# Circulating Hepcidin-25 Is Reduced by Endogenous Estrogen in Humans

**DOI:** 10.1371/journal.pone.0148802

**Published:** 2016-02-11

**Authors:** Mikael Lehtihet, Ylva Bonde, Lena Beckman, Katarina Berinder, Charlotte Hoybye, Mats Rudling, John H. Sloan, Robert J. Konrad, Bo Angelin

**Affiliations:** 1 Department of Endocrinology, Metabolism and Diabetes, Departments of Medicine and Molecular Medicine and Surgery, Karolinska Institutet at Karolinska University Hospital, S-141 86 Stockholm, Sweden; 2 Molecular Nutrition Unit, Center for Innovative Medicine, Department of Biosciences and Nutrition, Karolinska Institutet at Karolinska University Hospital, S-141 86 Stockholm, Sweden; 3 Lilly Research Laboratories, Eli Lilly & Co, Indianapolis, Indiana, United States of America; Lady Davis Institute for Medical Research/McGill University, CANADA

## Abstract

**Objective:**

Hepcidin reduces iron absorption by binding to the intestinal iron transporter ferroportin, thereby causing its degradation. Although short-term administration of testosterone or growth hormone (GH) has been reported to decrease circulating hepcidin levels, little is known about how hepcidin is influenced in human endocrine conditions associated with anemia.

**Research design and methods:**

We used a sensitive and specific dual–monoclonal antibody sandwich immunoassay to measure hepcidin-25 in patients (a) during initiation of in vitro fertilization when endogenous estrogens were elevated vs. suppressed, (b) with GH deficiency before and after 12 months substitution treatment, (c) with hyperthyroidism before and after normalization, and (d) with hyperprolactinemia before and after six months of treatment with a dopamine agonist.

**Results:**

In response to a marked stimulation of endogenous estrogen production, median hepcidin levels decreased from 4.85 to 1.43 ng/mL (p < 0.01). Hyperthyroidism, hyperprolactinemia, or GH substitution to GH-deficient patients did not influence serum hepcidin-25 levels.

**Conclusions:**

In humans, gonadotropin-stimulated endogenous estrogen markedly decreases circulating hepcidin-25 levels. No clear and stable correlation between iron biomarkers and hepcidin-25 was seen before or after treatment of hyperthyroidism, hyperprolactinemia or growth hormone deficiency.

## Introduction

Hepcidin-25 plays a key role in the regulation of iron metabolism in humans by controlling the absorption of iron from the intestine [1]. It is mainly synthesized in the liver as an 84 amino acid preprohormone which is converted to an active 25-amino acid peptide hormone detectable in serum and urine [2, 3]. Hepcidin-25 binds to the iron transporter ferroportin, present on the basolateral plasma membrane of intestinal enterocytes [4, 5]. Through a mechanism that is still incompletely understood, this binding causes the internalization and subsequent degradation of ferroportin, which leads to enterocytes becoming unable to transport iron across their basolateral plasma membranes [2, 3].

Hepcidin-25 was originally described as an antimicrobial protein belonging to the defensin group [6, 7]. However, the increased knowledge of the unique functions of Hepcidin-25 in iron metabolism and its effects on erythropoiesis has substantially influenced our thinking as regards not only iron overload diseases but also of anemia [8], and treatment in testosterone deficiency [9]. Mammalian iron homeostasis is concertedly regulated through hepcidin and ferroportin that fundamentally govern iron absorption, transport, storage and utilization [2, 3, 5]

The endocrine system plays a major role in the regulation of erythropoiesis and this may in part be through effects on Hepcidin-25. For example, we have previously shown that 3 weeks of GH treatment in healthy subjects decreased hepcidin-25 concentrations by approximately 65%, presumably by stimulating erythropoiesis [10]. In addition, a decreased level of hepcidin-25 has been shown following testosterone administration in healthy young and old men, and in older men with a high burden of chronic diseases [9, 11]. This effect seems to be independent of dihydrotestosterone [12]. Hypopituitarism is strongly associated with anemia [13] due to impaired red cell production. Improvement but not complete recovery of a decreased hemoglobin level was achieved by replacement treatment with hydrocortisone and/ or levotyroxine in men [13], indicating that additional factors such as GH and testosterone may be involved [14, 15]. Previous studies in mice have shown that testosterone suppresses HAMP gene transcription independently of the erythropoetin level [11]. In hyperthyroidism, a modest but significant anemia is seen in approximately one third of the patients [16], mimicking that associated with chronic disease and iron deficiency [17]. Normalization of hemoglobin levels following treatment of hyperthyroidism was accompanied by increases in mean corpuscular volume (MCV) and total iron-binding capacity (TIBC), while ferritin and erythropoietin (EPO) levels decreased [16]. Although there is limited information on the potential importance of estrogen in the regulation of iron metabolism in humans, recent data from animal experiments suggest that estrogen may influence hepcidin-25 in a significant way [18]**.**

In hereditary hemochromatosis, iron overload presents because of inappropriately reduced hepcidin-25 concentrations causing excessive iron uptake [19]. However, suppression of hepcidin-25 alone cannot fully explain the regulation of erythropoiesis associated with testosterone administration. This is because hemochromatosis patients with inactivating mutations in the hepcidin gene (*HAMP*), and mice with disruption of the hepcidin gene expression do not display a polycythemic phenotype [20, 21].

In order to further explore the potential importance of hormonal regulation of circulating hepcidin-25 in humans, we used a highly specific and sensitive sandwich ELISA assay [22] to study a set of endocrine conditions. In particular, we compared individuals before and after perturbations of estrogen, GH, thyroid hormone, and prolactin levels. Our findings indicate that changes in endogenous estrogen levels induced with gonadotropins influence the levels of circulating hepcidin-25, whereas no changes were seen in chronic GH deficiency, hyperthyroidism or hyperprolactinemia before or after restoration to physiological conditions.

## Materials and Methods

### Human samples

Serum samples collected in the course of prior studies [23–26] were stored at −70°C before analysis of hepcidin-25, C-reactive protein (CRP), ferritin, transferrin, TIBC, and hormone concentrations. Table 1 shows the characteristics of the study populations.

**Table 1:** Baseline characteristics of study participants.

Diagnosis	Gender (F/M)	Age (Range)	BMI (Range)
IVF stimulation	31/0	33 (25–38)	24,1 (18.7–31.3)
GH-deficiency	6/4	45 (26–62)/56 (54–59)	27.7 (21–34.8)/25.1 (20.5–28)
Thyrotoxicosis	16/4	48 (24–73)/37 (18–63)	23.7 (18–29.2)/21.5 (15.6–23.9)
Prolactinoma	8/6	36 (18–71)/44 (33–56)	25 (17.8–29.2)/27.5 (23.4–32.9)

Detailed information on the study protocols are given in references [23–26]. Data are expressed as median, min and max range.

The previous studies were in summary:

1. To induce controlled ovarian hyperstimulation: Endogenous estrogens were first suppressed using a gonadotropin releasing hormone agonist, buserelin (Suprecur®, Aventis Pharma, Frankfurt, Germany). After the menstrual bleeding, 2 weeks later, an estradiol (E_2_) measurement was carried out to verify suppression of estradiol. The response was followed up by an E_2_ measurement 6 days later, and by ultrasound scanning of the ovarian follicles 9 to 10 days after the first follicle-stimulating hormone injection [26].
2. To treat GH deficient patients. The diagnosis GH deficiency was verified by a peak GH response of <3 μg L^−1^ after stimulation using the insulin-induced hypoglycaemia and arginine stimulation tests. Patients were treated with conventional substitution with thyroxine, hydrocortisone, vasopressin or sex steroids for >2 years but not received previous GH treatment. Daily subcutaneous injections of GH were administered at bedtime and doses were then individually titrated to reach normalized insulin-like growth factor 1 (IGF-1) levels [25]. Serum samples was analyzed prior to and at 1 year of replacement with GH treatment.
3. Treatment of thyreotoxicosis. The diagnosis was based on serum levels of TSH, free thyroxin concentrations and thyroid antibodies. Seventeen patients were having Grave’s disease; 16 of these were treated with tiamazol (Thacapzol) and levothyroxine, and one patient received radioiodine treatment and subsequent levothyroxine treatment. One patient was diagnosed with toxic uninodular goiter and treated with radioiodine. Two patients were diagnosed with subacute thyroiditis with transient nodular thyrotoxicosis; they became euthyroid without medical treatment [24]. The time interval between the samplings (thyreotoxicosis vs. euthyroid) ranged between 4 and 25 weeks (mean ± SD, 14 ± 6 weeks).
4. For patients with hyperprolactinemia, inclusion criteria were elevated prolactin levels found on at least two occasions and magnetic resonance imaging of the hypothalamic-pituitary region confirming a prolactinoma. Eight women and six men were included. Patients were examined at base line and after six months of dopamine agonist therapy.

All participants had given their oral and written consent to participate in the respective studies. The local Ethics Committee in Stockholm approved the study design and the consent procedure.

### Hepcidin-25

Hepcidin-25 concentrations were measured with a high-sensitivity sandwich ELISA specific for hepcidin-25, as described previously [22]. In brief, a human hepcidin-25 Meso Scale Discovery (MSD^|Pr^) ELISA was performed by using streptavidin-coated and blocked wells that had been incubated for 1 h with biotinylated antihepcidin capture antibody (2 mg/L). After aspiration, the wells were washed 3 times with TBST [Tris-buffered saline (10 mmol/L Tris, pH 7.40, 150 mmol/L NaCl) containing 1 mL/L Tween 20]. Then, 100 μL hepcidin-25 calibrators (Peptides Insitute) (different hepcidin-25 concentrations in assay buffer, consisting of 50 mmol/L HEPES, pH 7.40, 150 mmol/L NaCl, 10 mL/L Triton X-100, 5 mmol/L EDTA, and 5 mmol/L EGTA) were added to the wells to generate a standard calibration curve. Serum samples were diluted with 20 parts assay buffer and added to their respective wells, and the ELISA plate was incubated for 1 h at room temperature. After aspiration, wells were washed 3 times with TBST, and 100 μL of a solution of conjugate antibody (ruthenium-labeled anti–hepcidin-25 detection antibody, 1 mg/L) diluted with 1000 parts TBST was added to each well for a 1-h incubation at room temperature. After aspiration, wells were washed 3 times with TBST, and the plate was developed with an MSD reader, which recorded ruthenium electrochemiluminescence.

### CRP, Ferritin, Transferrin, and TIBC

CRP concentrations were evaluated by immunoturbidimetry; this was done on a DxC/LX analyzer (Beckman Coulter Inc, Brea, USA). Serum ferritin was determined on Beckman Coulter DXI by chemiluminescent immunoassay. Serum iron and TIBC were measured by spectrometry on a Beckman Coulter DXC 800 (Beckman Coulter Unicel DXC systems). All samples were analyzed according to the manufacturer’s advice at the Department of clinical chemistry, Karolinska University Hospital.

### Tyroxine, Prolactin, Estradiol, and Insulin-Like Growth Factor-I (IGF-I)

Serum levels of free thyroxine (fT4) were measured using a modular analytics P170/P800 from Roche Diagnostics GmbH (Mannheim, Germany). Serum prolactin was measured using a commercial chemiluminescence immunoassay (Beckman Coulter Unicel, DXI). Serum was not screened for macroprolactin due to the high specificity of the prolactin method. Free estradiol was measured using routine clinical assays (detection limit, 0.15 ng/mL) as described previously [26]. Total serum IGF-I was determined by an in-house RIA after the separation of IGFs from IGFBPs by acid ethanol extraction and cryoprecipitation [27]. The detection level of the RIA was 3.0 mg/L. Cross-reactivity with IGFBP-2 and IGFBP-3 was less than 0.5 and 0.05%, [27].

### Statistical Analysis

Statistical analyses of hepcidin-25, CRP, ferritin, transferrin, TIBC and hormone concentrations were calculated using *Wilcoxon signed-rank test*. Correlations were tested by calculation of the Spearman rank correlation coefficient. Data are expressed as median, (lower and upper quartile). Significance level was set at p < 0.05. Statistical analyses were performed using Statistica, Statsoft version 10.0 (Tulsa, OK, USA).

## Results

### Endogenous estrogen reduces hepcidin-25 levels

Altogether 31 healthy females were studied during suppression and stimulation of endogenous estrogen levels as part of pretreatment for IVF (Table 2). There was a marked difference in estradiol levels between the two conditions with a more than 25-fold increase in the stimulated stage. Following estrogen stimulation, the levels of serum hepcidin-25 were reduced by almost 40% compared to the castration state (p < 0.01; Table 2). Whereas there were no correlations between hepcidin-25 and any indicator of iron metabolism in the estrogen-suppressed situation, hepcidin-25 correlated positively with iron (r = 0.38, p <0.05), TIBC (r = 0.44, p< 0.05), and ferritin (r = 0.8, p<0.0001) during estrogen stimulation. In contrast, CRP levels were increased in response to endogenous estrogen.

**Table 2:** Effect on hepcidin-25 level during initiation of in vitro fertilization using blood samples obtained when endogenous estrogens were low and high [26].

	Estradiol suppression, Median(lower and upper quartile)	Estradiol stimulated Median, (lower and upper quartile)	P value
Estradiol (ng/mL)	0.15 (0.15–0.27)	3.99 (1.62–19.5)	<0.001
Iron(uM/L)	15 (7–27)	15 (13–21)	ns
TIBC(%)	0.25 (0.1–0.37)	0.25 (0.18–0.33)	ns
Transferrin (g/L)	2.56 (2.06–3.32)	2,44 (2,32–2,75)	ns
CRP (mg/L)	0.69 (0.2–10.5)	1.6 (0.61–2.7)	<0.05
Ferritin (ug/L)	27 (9–45)	22.5 (13.5–31.5)	0.068
Hepcidin-25(ng/mL)	4.85 (0.56–12.77)	1.43 (0.78–5.03)	<0.01

Results for Hepcidin-25, CRP, ferritin, transferrin, TIBC and estrogen concentrations are expressed as median, (lower and upper quartile). Significance level was set at p < 0.05. For statistical calculation the *Wilcoxon signed-rank test* were used.

### GH substitution of GH-deficient patients does not influence hepcidin-25 serum levels

Patients with GH deficiency all had markedly reduced IGF-I levels that were normalized following treatment with GH for one year (Table 3). Despite this, there was no concurrent response in hepcidin-25 levels, or in any other metabolic parameter, following GH treatment.

**Table 3:** Results for Hepcidin-25 among GH-deficient patients studied prior to and during one years of replacement with GH [25].

	GH-deficiency, Median, (lower and upper quartile)	GH- treated, Median, (lower and upper quartile)	P value
IGF-1(ug/L)	86.5 (66–112)	195.5 (144–225)	< 0.01
Iron (uM/L)	16.5 (14–20)	20.5 (18–22)	ns
TIBC (%)	0.27 (0,25–0.37)	0.32 (0.27–0.39)	ns
Transferrin (g/L)	2.28 (2.26–2.47)	2.4 (2,21–2.66)	ns
CRP (mg/L)	3.15 (1.4–3.9)	1.7 (1.2–2.6)	<0.01
Ferritin (ug/L)	65 (45–215)	47 (40–160)	ns
Hepcidin-25 (ng/mL)	6.2 (4.04–15.25)	13.15 (7.05–16.1)	ns

Results for Hepcidin-25, CRP, ferritin, transferrin, TIBC and IGF-1 concentrations are expressed as median, (lower and upper quartile). Significance level was set at p < 0.05. For statistical calculation the *Wilcoxon signed-rank test* were used.

### Hyperthyroidism does not influence hepcidin-25 levels

Hyperthyroid patients had elevated fT4 and suppressed TSH levels (TSH data not shown) that were normalized following treatment (Table 4). There was no difference in hepcidin-25 levels between the hyperthyroid and the euthyroid state. Ferritin was increased, while transferrin and CRP were reduced during hyperthyroidism.

**Table 4:** Results for Hepcidin-25 during hyperthyroid vs euthyroid state [24].

	Hyperthyroid Median, (lower and upper quartile)	Euthyroid Median, (lower and upper quartile)	P value
fT4 (pmol/L)	42.5 (34–68,5)	19 (13.5–22)	<0.001
Iron (uM/L)	19.5 (15–26)	17.5 (14.5–21.5)	ns
TIBC (%)	0.35 (0.25–0.49)	0.25 (0.20–0.36)	0.05
Transferrin (g/L)	2.13 (1.96–2.55)	2.50 (2.32–2.84)	<0.001
CRP (mg/L)	0.67 (0.44–1.08)	0.75 (0.52–1.85)	<0.05
Ferritin (ug/L)	86 (39–133)	36 (27–75)	0.001
Hepcidin-25 (ng/mL)	7.52 (4.53–19.05)	5.47 (2.44–13.32)	ns

Results for Hepcidin-25, CRP, ferritin, transferrin, TIBC and free thyroxin concentrations are expressed as median, (lower and upper quartile). Significance level was set at p < 0.05. For statistical calculation the *Wilcoxon signed-rank test* were used.

Treatment of hyperthyroidism decreased levels of ferritin significantly, and increased transferrin levels, see Table 4. There was no significant difference in hepcidin-25 serum levels in the hyperthyroid and euthyroid conditions; however, the CRP level was significantly higher in the euthyroid state.

### Prolactin does not influence circulating hepcidin-25

Patients with prolactinoma were studied before and after 6 months of therapy with dopamine agonist therapy, resulting in normalized prolactin levels (Table 5). Also in this case, there was no difference in hepcidin-25 levels between the two situations.

**Table 5:** Results for Hepcidin-25 before and after six month treatment of prolactinoma with dopamine agonist therapy [23].

	Untreated prolactinoma Median, (lower and upper quartile)	treated prolactinoma Median, (lower and upper quartile)	P value
Prolactin (ug/L)	1066.0±674.75	9.16±1.05	<0.0001
Iron (uM/L)	21 (17–22)	14.0 (11.0–18.0)	ns
TIBC (%)	0.32 (0.25–0.38)	0.21 (0.17–0.28)	ns
Transferrin (g/L)	2.59 (2.3–3.08)	2.52 (2.41–2.82)	ns
CRP (mg/L)	1.0 (0.38–1.5)	0.69 (0.29–1.20)	ns
Ferritin (ug/L)	52.5 (32.5–79.5)	36.0 (24.5–66.5)	ns
Hepcidin-25 (ng/mL)	8.52 (4.8–20.95)	4.31 (2.84–7.40)	ns

Results for Hepcidin-25, CRP, ferritin, transferrin, TIBC and prolactine concentrations are expressed as median, (lower and upper quartile). Significance level was set at p < 0.05. For statistical calculation the *Wilcoxon signed-rank test* were used.

## Discussion

Circulating hepcidin-25 is a major physiological regulator of iron metabolism and haematopoesis. In order to explore the possible influence of a set of human hormones on hepcidin-25 levels, we studied several endocrine perturbations in well-characterized clinical conditions. Of particular relevance is the fact that we were able to compare situations of over- or under-function in the same individuals, thereby eliminating the influence of inter-individual variation in hepcidin-25 levels, which is known to be wide [22, 28]. Our major new finding is that variation in endogenous estrogen levels was associated with a marked response in hepcidin-25. Thus, high levels of estradiol induced by gonadotropin stimulation resulted in suppressed serum levels of hepcidin-25.

Premenopausal non-pregnant women with regular menstruation cycles are often deficient in iron [29]; the opposite is a frequent finding in postmenopausal women that also have higher levels of iron [18] but also a much lower recommended daily iron intake [30]. Iron levels are also increased in women who take oral contraceptives [29]. This effect may however be due to smaller volume of their menses. Estrogen replacement therapy in postmenopausal women has been reported to have no effect on serum ferritin or hemoglobin level. [31]. However, the potential role of estrogens on iron homeostasis is not clearly understood. In a previous study on mice and cell culture, estrogen contributed to maintaining iron metabolism by regulation of ferroportin via an estrogen response element (ERE) to maintain the intracellular iron level [32]. The healthy women included in our study were evaluated during initiation of IVF, when samples could be obtained when endogenous estrogens were low and high, respectively. Compared to the other groups of patients, they had a lower serum ferritin level, and to a lesser degree also lower levels of serum iron and TIBC. The healthy premenopausal women did also have lower levels of hepcidin compared to the other endocrine conditions we studied. Regular menstruation cycles in our cohort of studied females could explain the low basal levels of hepcidin compared with previous studies on testosterone in men [9, 11].

In male mice castration significantly increased hepcidin levels, whereas hepcidin mRNA levels were unchanged in ovariectomized female mice [33]. Thus, the effect of testosterone on hepcidin production seems comparatively more pronounced than that seen for estrogens. The possibility that the follicle-stimulating hormone stimulation used in our present model might exert some of its effect on hepcidin levels through stimulation of testosterone secretion from the ovaries [34] cannot be excluded.

It has previously been shown that premenopausal women also have a lower hepcidin-25 level at the basal state [28] which is concordant with our results. During estrogen stimulation, hepcidin-25 levels decreased significantly, but without any concordant increase of serum iron or serum ferritin. This may be related to a diminished iron intake during the stimulation period, or to that the study period was too short to allow the detection an iron increase [35]. The conflicting results on iron hemostasis in our study could also be reflected by the fact that the biological effect induced by estrogen deficiency needs at least 4–6 weeks to be reflected in the systemic iron hemostasis according to ovariectomized models [36]. In a recent study, a novel mechanism by which estradiol could increase iron absorption and iron release from storage cells was proposed [18]. In brief, the authors described a functional ERE half-site in the human hepcidin promoter, and they observed that 24 h after estradiol treatment, hepcidin mRNA decreased both *in vitro* in human liver cells and *in vivo* in mice. An estrogen antagonist (ICI 182,780) prevented the estradiol-induced decrease and, independent of estradiol treatment, caused an increase in hepcidin mRNA, indicating that the estrogen receptor may have a ligand-independent effect on hepcidin expression. Our *in vivo* data support such a mechanism of estrogenic action on hepcidin-25 which in part seems to be independent of the moderate iron deficiency seen in our group of premenopausal, non-pregnant women.

The small increase in the CRP level during initiation of IVF did not correlate with estrogens levels (data not shown), supporting previous studies [37, 38] concluding that estradiol does not correlate with CRP levels. Hepcidin expression is increased in inflammatory states. In our study there was a small but significant increase of CRP during estradiol suppression but no significant increase of ferritin or significant decrease of transferring during stimulation. This supports the concept that estradiol per se, and not reduced inflammatory activity, decreased hepcidin levels.

We have previously shown that daily administration of supraphysiological doses of GH to healthy volunteers for 3 weeks caused a sustained and significant suppression of hepcidin-25 concentrations [10]. In this situation, there is probably stimulation of erythropoesis [39]. However, in the GH-deficient patients reported here, who were treated to normalization of their initially low IGF-I levels, no significant effect on hepcidin-25 was seen. The fact that their initially normal ferritin levels were unchanged also argues against an important physiological role of GH in the regulation of hepcidin-25 in humans. The observed increase in hepcidin-25 following supraphysiological doses of GH [10] may instead reflect an acute-phase response, similar to the induction of circulating FGF21 that is observed at high doses but not following sustained substitution in GH-deficient patients [25].

In the present work, ferritin levels were increased, and transferrin levels decreased, in the hyperthyroid state, without significant changes in hepcidin-25 or iron. This indicates that thyroid hormone does not primarily influence the hepatic production of hepcidin-25. We also explored whether there was any major influence of excess prolactin on hepcidin-25, but were not able to find any substantial change when this was normalized.

In conclusion, while testosterone *per se* [9, 11] has been shown to influence circulating hepcidin-25 levels in humans with suppressed gonadotropins, our current study is the first to show that estrogen can also directly affect hepcidin-25 levels. This mechanism may at least partly be responsible for the known effects of estrogens on iron homeostasis and erythropoiesis. Further studies should be performed in order to gain insight to the possible molecular mechanisms of these potentially important and different regulatory elements in hematological function.

## Abbreviations

BMI: Body Mass Index
GH: Growth Hormone
IVF: in vitro fertilization
Hb: Hemoglobin
MCV: mean corpuscular volume
IGF-1: insulin-like growth factor 1
IL-6: Interleukin 6
TNF-α: tumor necrosis factor alpha
